# Passive surveillance assesses compliance with COVID-19 behavioural restrictions in a rural US county

**DOI:** 10.1017/S0950268821002107

**Published:** 2021-09-16

**Authors:** Christina L. Faust, Brian Lambert, Cale Kochenour, Anthony C. Robinson, Nita Bharti

**Affiliations:** 1 Department of Biology, Center for Infectious Disease Dynamics, Eberly College of Science, Pennsylvania State University, University Park, PA, USA; 2 Department of Geography, GeoVISTA Center, College of Earth and Mineral Sciences, Pennsylvania State University, University Park, PA, USA

**Keywords:** Behavioural restrictions, contacts, movement, rural health, SARS-CoV-2

## Abstract

Following the emergence of SARS-CoV-2, early outbreak response relied on behavioural interventions. In the USA, local governments implemented restrictions aimed at reducing movements and contacts to limit viral transmission. In Pennsylvania, restrictions closed schools and businesses in the spring of 2020 and interventions eased later through the summer. Here we use passive monitoring of vehicular traffic volume and mobile device-derived visits to points of interest as proxies for movements and contacts in a rural Pennsylvania county. Rural areas have limited health care resources, which magnifies the importance of disease prevention. These data show the lowest levels of movement occurred during the strictest phase of restrictions, indicating high levels of compliance with behavioural intervention. We find that increases in movement correlated with increases in reported SARS-CoV-2 cases 9–18 days later. The methodology used in this study can be adapted to inform outbreak management strategies for other locations and future outbreaks that use behavioural interventions to reduce pathogen transmission.

## Introduction

**Fig. 1.**
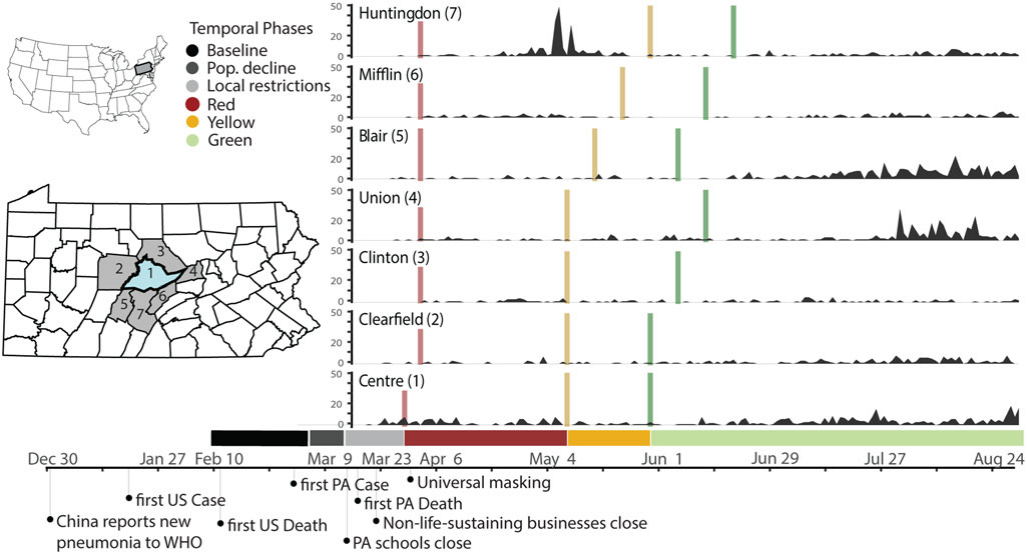
Timeline of policy interventions and cases in Centre County and adjacent counties. Left inset maps: location of Pennsylvania in the USA with locations of Centre County (blue) and surrounding counties (grey). Right: Daily case reports by county, colour bars indicate the onset of restriction phases. Below: Colours for all temporal phases for Centre County. Bottom: Timeline of relevant events.

Severe Acute Respiratory Syndrome Coronavirus 2 (SARS-CoV-2), the virus that causes Coronavirus Disease 2019 (COVID-19) was first detected in Wuhan, China, in December 2019 [1]. The WHO declared that COVID-19 was a pandemic in March 2020 (Fig. 1) [2]. COVID-19 is a respiratory disease in humans and clinical presentation can include a variety of secondary symptoms, including gastrointestinal and neurological indications, that range from mild to severe or fatal [3, 4]. The virus is spread by respiratory droplets, and transmission can occur through close contacts [5]. Infectious individuals can be asymptomatic or mildly symptomatic [3]. Similar to early responses to other novel or emerging pathogens, targeted pharmaceutical interventions for SARS-CoV-2 were initially limited. Large-scale outbreak management efforts focused on behavioural interventions to reduce transmission [6–8]. Assessing changes in movement levels, contacts and potential transmission events can help establish early warnings, implement adaptive control strategies and disseminate preventative public health messaging to slow transmission.

There are many ways to measure human populations and movement that are important for disease transmission. Data resolution varies across spatiotemporal scales, from targeted individual surveys and censuses [9] to large-scale passive surveillance data generated by satellites [10] and mobile devices [11]. Estimates of human movement and contacts for disease management have included tracking currency [12], commercial air traffic to model long distance flows [13], anthropogenic illumination to quantify seasonal or long-term population changes [10, 14, 15], and mobile devices for mobility traces [7, 16, 17]. Privately owned mobile device data are highly confidential and cannot be shared with policy makers. Researchers can obtain deidentified device data from third parties, which may be expensive or rely on opaque, proprietary algorithms. To overcome limitations on data sharing and increase replicability, we pair aggregated mobile device-derived data [18] with publicly available traffic camera images to measure human movement.

Early in the pandemic in the USA, county-level reporting showed that large cities and well-connected metro areas were hit the hardest [19]. As the outbreak progressed, it predictably moved into smaller towns and rural areas across the country [20]. Connectivity *between* locations loosely determines how early an infectious agent will arrive in a place and movement *within* a location determines how rapidly it can spread locally [21]. To reduce transmission, many states within the USA introduced large-scale quarantines targeting connectivity and movement. Understanding movement and pathogen transmission in rural areas is important because rural movement patterns differ from those found in urban areas [22, 23]. Additionally, residents in rural areas experience barriers to health care access that are different from barriers in urban areas [24, 25]. Rural residents in the USA also have lower median household incomes and often rely on under-resourced health care services [26].

Centre County is located in a rural valley in central Pennsylvania. It is home to The Pennsylvania State University's (PSU) University Park (UP) campus, the largest campus of the state's largest public institute of higher education. PSU is one of 112 land grant universities in the country. These institutions were established with a focus on agricultural education and improvement and, like PSU, many are still surrounded by suburban and rural areas. In 2019, the median US household income was $65 712 while the median Centre County, PA, household income was about 8% lower – $60 706 [27].

Prior to COVID-19, Centre County had 12 intensive care unit beds, which increased to 24 during the pandemic [28]. Testing capacity remained limited throughout the USA in 2020, including in Centre County. Test results were returned with delays of days or weeks, which hindered contract tracing efforts [29] and broad-scale restrictions remained necessary.

Centre County, Pennsylvania, first implemented policies to minimise virus spread and transmission in March, in the form of travel restrictions and stay at home orders (Fig. 1; Table 1). We monitored indicators of human moment in response to COVID-19 restrictions and the resulting outbreak trajectories from March through mid-August 2020, focusing on times when the university was not holding in-person classes and most students were not residing in the county. Our analysis begins 6 weeks prior to the strictest restrictions and ends during 2020's most lenient county-level restrictions.

**Table 1.** Details of Pennsylvania COVID-19 restriction phases (dates for Centre County)

Restriction phase	Length of phase	Work and congregate settings	Social settings
Red	40 days (28 March–7 May)	Life-sustaining businesses only Schools closed for in-person teaching Most childcare closed	Stay at home order Large gatherings prohibited Travel limited to life-sustaining purposes Restaurants/bars limited to carry-out and delivery
Yellow	20 days (8 May–28 May)	Telework where feasible Businesses with in-person operations must follow safety guidelines Schools closed for in-person teaching Some childcare open with safety orders	Full stay at home restrictions lifted In person retail allowed, but curbside/delivery preferred
Green	78 days (29 May–14 August for this study; though restrictions were in place until 12 December)	Businesses reopen with CDC and PA Department of Health (DOH) guidelines (reduced capacity, distancing, mask wearing)	Gyms, spas, entertainment reopen pending guidelines Aggressive mitigation orders lifted (individuals must follow CDC and PA DOH guidelines)

We measured two proxies for movement within Centre County: vehicle volumes during the pandemic from publicly accessible traffic cameras and two years of mobile device-derived visits to points of interest (POI) from SafeGraph [30]. Traffic volumes provide a good indication of movement in rural areas, which lack public transit and require residents to rely on private vehicles, but likely underrepresent movement in denser, pedestrian-heavy areas. Mobile device data likely under-sample the elderly and residents in areas with poor network coverage or speed but are likely to adequately represent movement in the pedestrian-heavy areas on and surrounding university campuses. An additional advantage of SafeGraph's ongoing passive surveillance is a long period of observation that precedes the pandemic.

We compared movement across restriction phases to measure uptake and compliance of behavioural interventions. We also tracked the daily incidence of confirmed SARS-CoV-2 cases to assess the effectiveness of the intervention measures. Overall, we found that compliance was high and movement only increased with lifting restrictions. We also found that cases increased as movement increased, suggesting behavioural interventions early in the pandemic were effective in minimizing SARS-CoV-2 transmission. The ongoing emergence of increasingly transmissible or virulent variants emphasises the importance of accurately measuring the uptake of behavioural interventions and estimating the resulting impact on transmission dynamics. Our methods are broadly applicable across rural areas that are using behavioural interventions to manage transmissible pathogens. These approaches can inform outbreak management strategies on the effectiveness of behavioural interventions going forward.

## Methods

### Study area

Centre County has an estimated population size of 162 385 residents [27] and approximately 38 000 of these residents are undergraduate students enrolled at PSU's UP campus [31]. Most undergraduate students do not reside in the county year-round, leading to declines in student population during the summer and winter breaks between semesters.

### Study period

We used traffic cameras and mobile device-derived visits to POI to detect changes in movement, with a focus on responses to restriction policies from 14 February 2020 to 14 August 2020 (Fig. 1). We defined six temporal phases that align with events and policies that impacted movement and behaviour at PSU's UP campus and across Centre County in 2020 (Table 1, Fig. 1):

1. *Baseline (14 February–6 March)*: before restrictions were in effect and while undergraduate students were on campus
2. *Population Decline (7 March–18 March)*: before local restrictions were in effect, after students left campus for spring break, and encompassing the transition to online instruction on 16 March
3. *Local Restrictions (19 March–27 March)*: Mandated closure of all non-essential businesses (no county-wide restrictions)
4. *Red (28 March–7 May)*: County-wide Red restriction phase
5. *Yellow (8 May 8–28 May)*: County-wide Yellow restriction phase
6. *Green (29 May 29–14 August)*: County-wide Green restriction phase

The calendar dates defining the Baseline (1), Local Restrictions (3) and Red (4) phases in 2020 occurred during the spring semester, which correspond to dates in 2019 when undergraduate students were in residence in Centre County. The Population Decline (2), Yellow (5) and Green (6) phases of 2020 coincided with times when semester classes were not in session in 2019 (spring break or summer session) and the student population in Centre County was drastically smaller. In 2020, students left campus during the Population Decline phase for spring break and were instructed not to return because the university implemented COVID-19 prevention policy to transition to online instruction from 16 March 2020.

To measure changes in movement through the restriction phases of 2020, we collected traffic data from 27 April 2020 to 14 August 2020. To measure changes in movement between years, we used SafeGraph mobile device-derived counts of visits to POI from 14 February 2020 to 14 August 2020 and the corresponding periods in 2019 [30]. Pennsylvania began reporting confirmed SARS-CoV-2 cases on 1 March 2020 [32].

### Traffic cameras

**Fig. 2.**
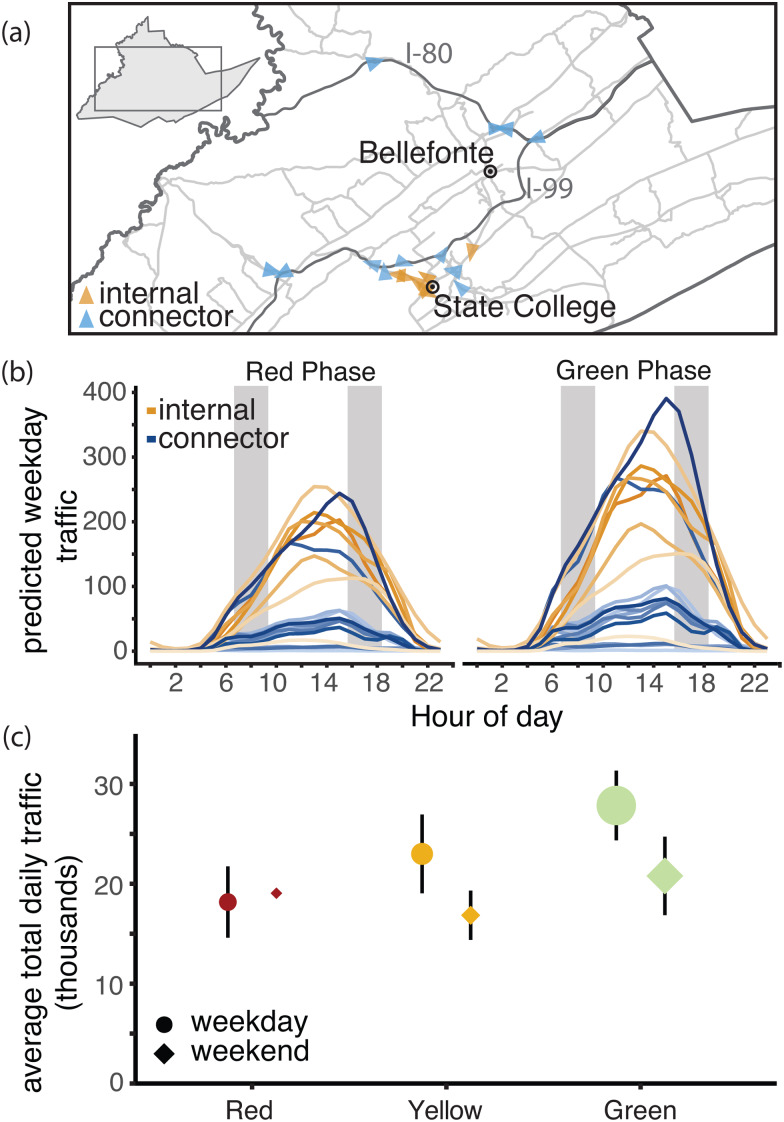
Traffic camera locations and volume throughout containment phases. (a) Locations of traffic cameras. Internal road traffic cameras (orange) were concentrated around the PSU campus and surrounding boroughs, the connector road cameras (blue) covered a larger spatial extent. The direction of each arrow points to the direction the image was captured. (b) Centre County hourly vehicle volumes during Red and Green phases. Traffic during all phases peaked between 12:00 and 18:00 h EST. Vehicle volume was significantly greater in the green phase with the largest increases on connector roads. Typical rush hours (07:00–09:00 and 16:00–18:00) are shaded in grey. (c) Traffic increased through each phase of easing of restrictions. The size of each point indicates the number of days of data collection. The smallest sample size collected was during weekends in the Red phase (2 days) and the largest number of observations was during weekdays in the Green phase (55 days).

We collected images from 19 traffic cameras across Centre County to quantify the number of vehicles on roads beginning on 27 April 2020 (Fig. 2a, Fig. S1). Twelve of these cameras surveil interstates, state highways or other roads that link towns in Centre County, which we refer to as ‘connector’ roads (Table S1). The remaining seven cameras capture ‘internal’ roads for travel within towns. Cameras produce 24 h live streams that are publicly accessible online but are not archived. We captured and stored images from these live streams approximately every 20 s. Using the Python package *cvlib* as a high-level interface to OpenCV [33, 34] and Google's open-source TensorFlow software stack [35], we identified and counted all vehicles in each image. Parked vehicles were identified and excluded from counts. The hourly number of vehicles captured by each camera was standardised by the number of images captured within that hour (see Supplementary Material for details).

We fit a series of generalised additive models (GAMs) to standardised and rounded hourly vehicle counts. GAMs are useful for fitting time series and can account for non-linearity between response and predictor variables. We modelled the hour of the day in local time as a cyclic cubic regression spline to account for non-independence of hourly traffic throughout each day. Using a cyclic spline allowed the same smoother to operate at the 12:00 h ‘start’ of a day and the 23:00 h ‘end’ of a day (Fig. S2). We also included the following additional predictor variables in models, with interactions between variables: day of week, weekday/weekend (binary variable), restriction phase (red/yellow/green), camera identity, number of lanes visible in camera image and road type (internal or connector road). We fit multivariate GAMs with a Poisson distribution using the package *mgcv* in R version 3.6.2 [36, 37]. Most of the traffic cameras experienced at least one short gap in image acquisition (Fig. S3). Missing hourly data, <6% of 49 248 camera-hours, were predicted using the best-fit GAM. The combined data (both observed and predicted) were used to estimate average daily vehicle traffic volumes and assess changes between restriction phases. From April to December of 2020, the 19 traffic cameras in Centre County captured a weekly mean of 167 465 vehicles (range 67 310–252 193). During the study period, these cameras captured a weekly mean of 166 835 vehicles (range 125 919–197 059 vehicles).

### Mobile device-derived data

SafeGraph, a data company, provides aggregated anonymised location data from numerous applications in order to provide insights about foot traffic in physical places, or POI, via the Placekey Community. These data include mobile device-derived counts of daily visits to POI (businesses, offices, university buildings, etc.) (Fig. S1). They sample visits from 45 million mobile devices across the USA to 3.6 million POI and their sampling correlates with census-derived population sizes [30]. From January to December of 2020, SafeGraph collected data in Centre County on 3 311 518 visit counts to 2188 POI from a weekly mean of 5183 mobile devices (range 2933–7831). During the study period corresponding to traffic data, SafeGraph captured 832 539 visits to 2129 POI from a weekly mean of 4148 devices (range 3568–5042) in Centre County.

We used data collected by SafeGraph in Centre County from 2019 and 2020. We used daily visit counts from early in 2020 before any COVID-19 restrictions were imposed, to establish expected visit counts during times when students were present and absent, in the Baseline and Population Decline phases, respectively. We used the differences to establish expected visit counts for comparison to observed visit counts while accounting for annual growth in SafeGraph data collection. These expectations allow us to quantify the changes in movements that were caused by the pandemic. To quantify the changes in movement through the restriction phases, we subtracted 2020 visit counts from 2019 visit counts. We aligned dates from 2019 to the corresponding academic periods in 2020 to account for differences in calendar dates (Table S1) [38]. We compared the differences in visit counts between years to the expected visit counts from early 2020 to quantify the observed difference in visit counts due to behavioural interventions.

### COVID-19 diagnostic testing results

We acquired total daily confirmed cases of COVID-19 in Centre County and the surrounding counties from the Pennsylvania Department of Health's version of the National Electronic Disease Surveillance System [32]. Cases are confirmed using CDC-approved diagnostic reverse transcriptase polymerase chain reaction tests, which detect viral RNA in active infections, and are reported on the day the test was completed. The case data included here extend to 27 August 2020, two weeks past the last date of inclusion for movement data and transmission events in this study. We included this 2-week lag to account for a 5–8 day incubation period [39] and 5.5–10.93 day delay from the onset of symptoms to case confirmation [3]. We used cross-correlation analyses [40] to identify significant time-lagged correlations between movement measurements and the 7-day moving average of reported cases. We used the 7-day moving average of incidence because large numbers of positive test results reported on a single day may be due to tests that were processed in batches, which do not necessarily reflect a single-day increase in cases. Correlation coefficients between the two time series are reported as autocorrelation functions (ACF) for a given temporal lag.

## Results

### Changes in traffic volume through restriction phases

Throughout the restriction phases, from 27 April 2020 forward, daily traffic volume followed a bell-shaped pattern, with minimal traffic at 02:00 h and peaks between 12:00 and 18:00 h (Fig. 2b, Fig. S2). The best-fit Poisson GAM to explain average vehicle counts (*y*) (Equation 1) is dependent on the time (subscript *i*) and camera identity (subscript *j*) of each observation. The full model includes splines (*f*) fit to hours of day (*h*) for each camera(*j*) with a mean dependent on the camera (*β*_4,*j*_), a binary weekend predictor (*w*), intervention phase (*p*), road type (connector or local) (*r*) and an interaction between intervention phase and road type (connector or local). The splines, intercept (*β*_0_) and the effects of each fixed effect (*β*_1−3_) were determined with generalised cross-validation. This model (Equation 1) explained 87.3% of deviance observed in vehicle traffic and was used to predict the missing 5.96% of hourly counts (Fig. S3).1
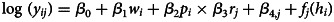


From April to August, Pennsylvania's county-level behavioural restriction phases eased twice and traffic volume increased significantly. We observed greater increases in numbers of vehicles during weekdays and on connector roads than during weekends or on local roads (Fig. 2b, c). During the Red phase, we calculated a daily weekday mean of 10 772 vehicles on internal roads and 7397 vehicles on connector roads (Table S3). After Centre County eased to the Yellow phase of restrictions, mean daily weekday traffic totals increased by 23.2% on internal roads (an increase of 2495 vehicles on weekdays) and 31.5% on connector roads (an increase of 2331 vehicles on weekdays). When the county transitioned from the Yellow to the Green phase of restrictions, we saw an additional 15.4% increase in daily weekday traffic on internal roads (an increase of 2042 vehicles on weekdays), and a 27.8% increase in vehicles on connector roads (an additional 2697 vehicles on weekdays).

### Changes in mobile device-derived visit counts between years and through 2020 restriction phases

**Fig. 3.**
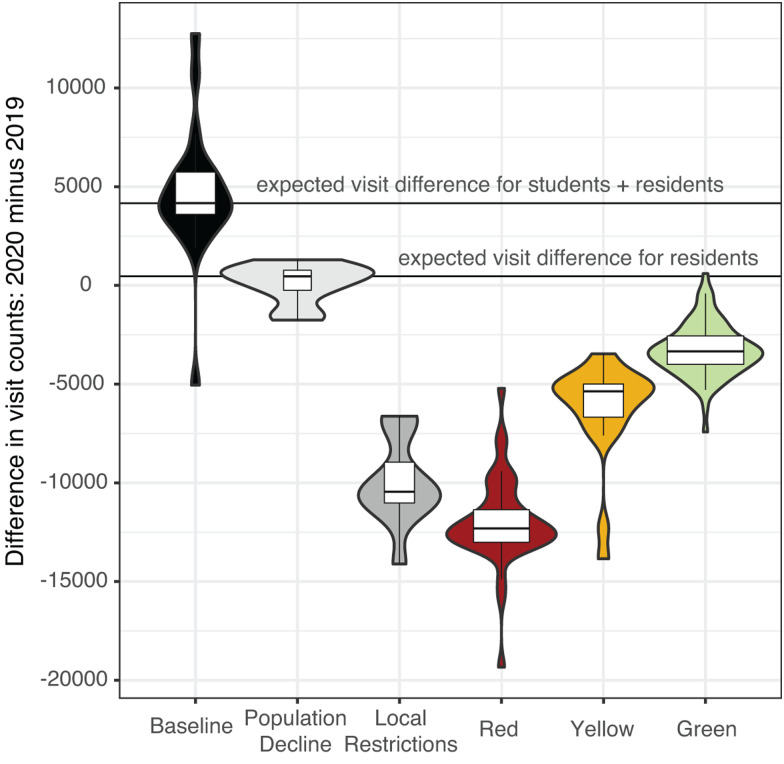
Aggregated mobile device visit counts. Summary of daily differences in visit counts between 2020 and 2019 within each phase. The width of boxplots within violin plots corresponds to the total number of days in each phase: minimum 8 days in Local Restrictions, maximum 78 days in the Red phase. The two horizontal lines indicate the median expected visit differential for 2020 compared to 2019 for students and residents combined (4167) and for full-time residents only (464).

Visit counts in 2020 were highest during the Baseline phase, when students were present (median daily visit count = 18 220) (Fig. S4). When compared to the same period in 2019, there were more visits in 2020 (median difference = +4613 in 2020) (Fig. 3). Visit counts in 2020 began to decrease after the Baseline phase, during the Population Decline phase in March. This decline occurred when students departed for spring break and was matched by declines due to spring break in 2019 (daily median differential of +464 visits in 2020). In 2019, visit counts increased immediately following spring break with the return of students, while in 2020 the visit counts continued to decline due to the university's pandemic protocol.

**Fig. 4.**
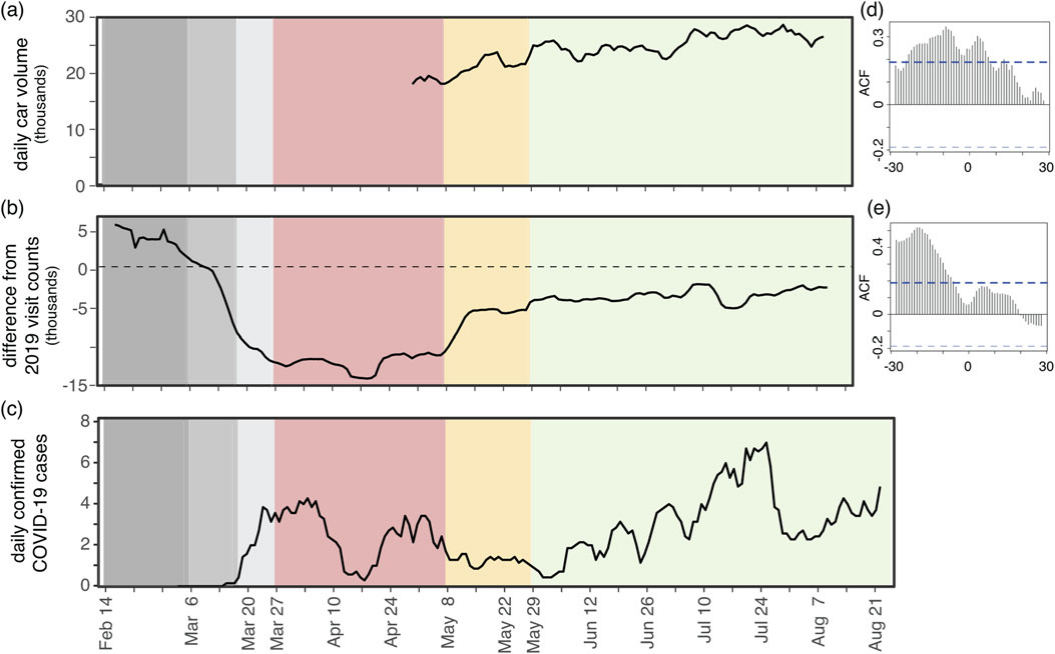
Estimated SARS-CoV-2 cases align with movement across phases. (a) Seven-day rolling mean of daily total traffic volume increases as restriction phases eased. (b) Seven-day rolling mean of daily differences in mobile device visit data between 2020 and 2019. (c) Seven-day rolling mean of daily confirmed COVID-19 cases. (d) Autocorrelation function of daily traffic volume compared to 7-day mean of cases. Significant positive lags and leads are above the dotted blue line. (e) Autocorrelation function of mobile device-derived visit differentials compared to 7-day mean of cases. Significant lags occur above the dotted blue line.

The largest negative differential between visit counts in 2019 and 2020 occurred during the Red phase, reflecting the combined effects of the strictest restrictions in Centre County and the absence of the student population in 2020 (Figs 3 and 4b). During the Yellow and Green phases of restrictions, 2020 visit counts increased, reducing visit differentials. However, 2020 summer visit counts never reached 2019 values or expected visit counts based on pre-pandemic behaviour from 2020, indicating ongoing and sustained reductions in movement, as measured by mobile devices in Centre County.

### Correlations between reported cases and movement

Daily COVID-19 case totals were low in Centre County throughout the summer of 2020 compared to urban and more populous counties in Pennsylvania [32]. Through 27 August 2020, Centre County confirmed a total of 448 cases, or 358.6 cases per 100 000 [32]. Daily incidence showed asynchronous outbreaks among neighbouring counties in central Pennsylvania (Fig. 1). In Centre County, increases in movement preceded reported cases (Fig. 4). We found a significant correlation between traffic and a 7-day moving average of cases, with a 9-day lag in cases showing the greatest correlation (ACF = 0.344, *P* < 0.05; Fig. 4a, d). Additionally, we found a significant lagged correlation between mobile device visits to POI and reported cases. There is a significant lag from 28 to 7 days prior to cases, but the greatest correlation occurs at a 19-day lag (ACF = 0.518, *P* < 0.05; Fig. 4b, e).

## Discussion

### Overview

Behavioural interventions were the primary large-scale public health tool available for preventing SARS-CoV-2 transmission throughout most of 2020. Behavioural interventions are most effective when implemented early and broadly. They are particularly important in areas where supportive care and medical resources are insufficient, such as underserved rural communities. Measuring levels of uptake of behavioural interventions is considerably more challenging than measuring uptake of pharmaceutical interventions but is critical for assessing the effectiveness of behavioural interventions and planning future outbreak response efforts. We used publicly available traffic volumes and mobile device-derived visits to POI to measure behavioural responses to COVID-19 restrictions in a rural county in central Pennsylvania. Overall, we found that movement measured by traffic volumes and mobile device-derived visits to POI increased as restrictions eased, indicating compliance. We also found that reported COVID-19 cases increased as movements increased with a 9- to 18- day lag, suggesting that compliance with behavioural interventions was effective in reducing SARS-CoV-2 transmission. These data sources can be used to monitor movement, compliance with behavioural interventions and adapt disease mitigation strategies in the future.

### Traffic and mobile devices

Data collected passively from traffic cameras measured movement in response to phased restriction policies. In Centre County, local roads provide access to essential businesses, which remained open throughout all restriction phases, while connector roads are used for travel between locations and may reflect movements to return to business, childcare and in-person work (i.e. during County Green phase). As restrictions eased, vehicle volumes increased on both local and connector roads, with greater increases on connector roads, particularly in the most lenient Green phase. Although changes in restriction phases were announced approximately one week in advance, traffic volumes increased on the date when restrictions officially eased, and increases were not observed earlier. These results suggest that Centre County residents largely complied with county-level restrictions. However, this region did not transition from a less restrictive phase to a more restrictive phase during the period studied so compliance with enforcing stricter restriction phases cannot be assessed.

Daily counts of SafeGraph mobile device-derived visits to POI and traffic volumes showed similar patterns: movement gradually increased from the Red phase to the Yellow phase and Green phase. SafeGraph data were also critical in providing movement data from 2020 for dates prior to the implementation of restrictions phases as well as for corresponding dates from 2019. Data prior to the Red phase are not available for traffic volumes because images are not archived and must be captured in real-time.

In the Red phase, mobile device visit counts decreased by 81% compared to the baseline period of 2020. While some of this is due to the absence of students following spring break, these visit counts are still lower than visit counts from 2020 during pre-pandemic times when students were not in residence as well as the corresponding dates for 2019. The Green phase brought an increase in daily visit counts but the median daily visit count was still 11% lower than expected based on 2020 visit counts that preceded pandemic restrictions and lower than the corresponding time period in 2019. With SafeGraph's longitudinal data, we were able to highlight compliance with restriction policies to calculate that visit counts in Centre County did not return to pre-pandemic levels during the summer of 2020, even as restrictions eased.

In Centre County, we collected data on more cars per day (weekday mean ranged from 18 169 (Red) to 27 734 (Green)) than mobile devices per day (weekday mean ranged from 3714 (Red) to 7470 (Green)) during the restriction periods, indicating the traffic data provide a better representation of the total county population than SafeGraph data. However, the differences in mobile device counts between the baseline and population decline periods of 2019 and 2020 show that SafeGraph data represent students better than residents. From March to August of 2019, the maximum mobile device visit counts from Centre County reflected known academic calendar events and university or community events near or on PSU's UP campus (Fig. S4). Although these two data sources showed similar movement trends, they provide different spatial and temporal coverage and it is likely they are measuring different subsets of the population in Centre County and different types of behaviours (Fig. S1). Including both data sources improves our understanding of compliance and reduces bias compared to focusing on a single measurement.

### Movement and cases

Increases in movement following easing restrictions likely led to increased contacts and contributed to the subsequent uptick in cases during the Green phase in Centre County. The bimodal pattern of infections in the summer most likely reflects increased SARS-CoV-2 transmission and not simply an increase in testing, which did not increase appreciably during this period and increased only in a monotonic direction [32]. We demonstrate a significant lag between movement, measured by traffic and SafeGraph data, and reported cases. Traffic data show both significant lags and leads with reported COVID-19 cases. This is likely because the period during which traffic data were collected included only increases in movement and no decreases and occurred during the initital phase of the outbreak. SafeGraph data, which covered periods of declining and increasing movement only showed significant lags with cases. The significant lags between movement and cases highlight that movement underlies increases in transmission and precedes increases in reported cases by about 2 weeks. Confirmed cases are reported on the day the test is completed and no other dates related to cases are publicly available (onset of symptoms, sample collection, etc.). We would prefer to use the date of sample collection for confirmed cases, and this is a limitation of this study. Instead, we assume consistent delays between collection and result throughout this period. We show a link between confirmed COVID-19 cases and movement that provides actionable metrics for proactive policy, preventive actions and modifying behavioural restrictions.

### Rural health

Many methods that are commonly used to track population movements are more effective in urban areas than in rural areas. Some methods rely on high smartphone usership and wireless network penetration or widespread and equitable access to high-speed Internet. Satellite radiance data have been successfully used to measure changes in economic and human activity in urban centres during COVID-19 lockdowns [41, 42], but due to high numbers of cloud days and small, sparse settlements, we could not measure rural population dynamics with serial satellite imagery in this setting. Aggregated cell phone data have also been used to map movements between dense population centres [7, 43], and this study demonstrates its utility within a rural population. However, we expect that any single data source cannot adequately represent a population, particularly a rural settlement. We intentionally used multiple, independently collected datasets to measure local movement patterns.

Overall, urban populations also have greater access to health care, despite inequities within populations. They also have better-resourced, higher capacity health care centres when compared to rural areas throughout the USA, which improves diagnostics and access to testing, as seen for COVID-19 [25]. Because of age structure and limited health care infrastructure, certain rural populations were highlighted as particularly vulnerable to COVID-19 [44, 45]. Small towns and rural areas experienced delayed SARS-CoV-2 introductions and lagged local outbreaks compared to urban centres [46]. Yet, statewide regulations largely responded to urban outbreak fluctuations, which were not synchronised with rural outbreaks. When paired with effective federal response and relief, local oversight can most effectively serve outbreak response, management and planning efforts.

## Conclusion

Early responses to the SARS-CoV-2 pandemic in the USA were managed by local governments implementing policies at state and county levels with insufficient resources or enforcement authority. COVID-19 spread widely through the nation's big cities and small towns. Moving forward, it will be important to monitor local outbreaks and movement to design and implement locally responsive interventions. In addition to pharmaceutical interventions, measuring local population movements through passive approaches can help estimate uptake of behavioural interventions and adapt policies that target transmission prevention. Monitoring local movements and contacts is necessary to assess the effectiveness of behavioural interventions intended to reduce pathogen transmission. Informed current and future uses of movement restrictions can help avoid overwhelming health care capacity, particularly in rural areas.

## Data Availability

All data used for this study are available from publicly available sources and are attributed in the methods section with the exception of SafeGraph data. Academic researchers must register to receive access to SafeGraph data at no charge for non-commercial purposes only here: https://www.safegraph.com/academics. No additional data have been generated by this study. Code to process raw data, generate models and figures is available at: https://github.com/bhartilab/covid_movement_centre

## Supplementary material

For supplementary material accompanying this paper visit http://dx.doi.org/10.1017/S0950268821002107. click here to view supplementary material
