# Supplementary material for: Passive surveillance assesses compliance with COVID-19 behavioural restrictions in a rural US county
Source: Epidemiol Infect. 2021 Sep 16;149:e211. doi: 10.1017/S0950268821002107 (PMC8485031; doi:10.1017/S0950268821002107)
Supplement: Supplementary file 1 [file S0950268821002107sup001.docx]

***Passive surveillance assesses compliance with COVID-19 behavioral restrictions in a rural US county***

Christina L Faust, Brian Lambert, Cale Kochenour, Anthony C. Robinson, Nita Bharti

**SUPPLEMENTARY MATERIAL**

**Supplementary Methods**

I. Detailed timeline of events

II. Details of traffic camera acquisition, standardization, and cleaning

**Supplementary Tables**

Table S1. Traffic camera locations and descriptions

Table S2. Calendar dates for phases between 2020 and 2019 for SafeGraph data

Table S3. Summary of daily traffic data

**Supplementary Figures**

Figure S1. Camera

Figure S2. Splines fit to hourly traffic volume by individual cameras

Figure S2. Observed and predicted of hourly count data per camera through time

Figure S3. Visit counts from SafeGraph for 2019 and 2020

**Supplementary Methods**

**I. Detailed timeline of events**

Figure 1 in the main text details many of the key disease and policy events that are relevant to changes in policies and movement at the Penn State University Park campus and throughout Centre County. We provide more detail and citations for information in Figure 1 to give additional context to the policies and messaging from March through August of 2020.

**Timeline of important disease and policy events local study periods:**

Dec 31, 2019: China reports to WHO new pneumonia

Jan 20, 2020: 1st case in US

[BASELINE PERIOD: Feb 18 – March 6]

February 12: first death in US (Santa Clara, CA, retrospectively identified)[1]

March 6: first 2 reported cases of COVID-19 in PA

[LOCAL POPULATION DECLINE: Feb 1 – March 6]

March 9: PSU spring break begins[2]

March 11: WHO declares COVID-19 a pandemic[3]; PSU announces no resident instruction following spring break[4]

March 16: PSU begins remote instruction

March 19: all non-life-sustaining businesses ordered to close statewide by PA Governor[5]

March 21: enforcement of closure of non-life-sustaining businesses statewide[5]

[RED PHASE: Feb 1 – March 6]

March 28: Centre County receives stay at home order from PA Governor [6]

April 3: PA Governor calls for “universal masking”

April 9: PA schools officially closed through end of academic year

May 1: announcement from PA Governor that on May 8 PA will lift some restrictions in 24 counties in the Northcentral and Northwest health districts; all counties will remain in “red” until May 8, these counties will move to “yellow” on May 8 [6]

[YELLOW PHASE: Feb 1 – March 6]

May 8: PA lifts some restrictions in 24 counties in the Northcentral and Northwest health districts as determined by PA Governor in moving from “red” (Stay-at-home) to “yellow” (aggressive mitigation) [6]

May 28: US death toll from COVID-19 passes 100,000

[GREEN PHASE: May 29 – August 14]

May 29: Some of Northcentral and Northwest health districts (Fig S2) move from “yellow” to “green”

June 30: First PSU student death from COVID-19

July 1: Order of face coverings in public places in Pennsylvania [7]

**II. Details of traffic camera acquisition, standardization, and cleaning**

We captured images every 20 seconds,from each of 19 cameras. To standardize variation in images captured per hour, with variation due to timing mismatches or missing images (see below), the sum of vehicles counted each hour is divided by the number of images captured each hour and scaled by the number of expected images if we captured one image per minute. This avoids overcounting cars at traffic lights but likely underestimates total vehicle volume on interstates. Therefore, count data should be interpreted as relative increases rather than absolute.

Occasionally, live stream traffic cameras would fail to capture images correctly, resulting in missing images. The standardized hourly counts were cleaned to remove strings of zeros or integers, which were the result of frozen images or occasionally, parked cars. None of the cameras capture legal parking zones when they are in the correct position. Incorrect vehicle counts from missing or erroneous images were replaced with NAs and predicted with the best fit generalized additive model (see Methods in Main Text; Fig S2).

**Supplementary Tables**

**Table S1. Traffic camera locations and descriptions**

| **ID** | **Latitude** | **Longitude** | **Ownership** | **Number of lanes** | **Road Type** | **Description** |
| --- | --- | --- | --- | --- | --- | --- |
| CAM02001CCTV2 | 40.810961 | -78.075259 | PennDOT (Pennsylvania Department of Transportation) | 5 | connector | Port Matilda US-322 By-Pass Westbound |
| CAM02002CCTV3 | 40.817144 | -77.939841 | PennDOT | 7 | connector | Grays Woods |
| CAM02003CCTV4 | 40.828613 | -77.840339 | PennDOT | 6 | connector | I-99/US-322 Interchange |
| CAM02005CCTV9 | 40.955477 | -77.773749 | PennDOT | 3 | connector | Milesburg Interchange West I-80 Exit 158 Eastbound / Alt US-220 |
| CAM02006CCTV10 | 40.956061 | -77.766277 | PennDOT | 3 | connector | Milesburg Interchange East I-80 Exit 158 Westbound / PA-150 |
| CAM02007CCTV13 | 40.795773 | -77.820937 | PennDOT | 4 | connector | US-322 E/O PA-26 |
| CAM02009CCTV7 | 40.944571 | -77.720918 | PennDOT | 2 | connector | I-80 Eastbound Exit 161 Bellefonte Interchange (I-80 & I-99) |
| CAM02010CCTV11 | 40.80388 | -78.063664 | PennDOT | 6 | connector | Port Matilda I-99 Exit 61 Median |
| CAM02020CCTV24 | 40.829048 | -77.804817 | PennDOT | 6 | internal | Benner Pike |
| CAM02028CCTV32 | 40.812073 | -77.9225 | PennDOT | 6 | connector | Atherton and Valley Vista |
| CAM02033CCTV38 | 41.022703 | -77.933981 | PennDOT | 4 | connector | Snowshoe I-80 Exit 147 |
| CAM02037CCTV43 | 40.807936 | -77.895204 | PennDOT | 5 | internal | Atherton and Vairo |
| CAM02038CCTV44 | 40.805031 | -77.886886 | PennDOT | 5 | internal | Atherton and North Hills |
| CAM02039CCTV45 | 40.796522 | -77.872549 | PennDOT | 5 | internal | Atherton and Park |
| CAM02040CCTV46 | 40.791787 | -77.864971 | PennDOT | 4 | internal | Atherton and W.College |
| CAM02042CCTV49 | 40.790894 | -77.863812 | PennDOT | 4 | internal | Atherton and W.Beaver |
| CAM02046CCTV52 | 40.817953 | -77.900271 | PennDOT | 4 | connector | I-99 at Exit 71 (Toftrees) |
| collegeTwp | 40.811564 | -77.830719 | State College Township | 4 | connector | College Township Traffic camera |
| parkArboretum | 40.804483 | -77.864219 | Penn State University | 3 | internal | Penn State University Park Ave. |

**Table S2. Calendar dates for phases between 2020 and 2019 for SafeGraph data.** The timing of Spring Break and Commencement at Penn State determine large scale movement events in State College and surrounding areas in Centre County. The timing of these events was matched across 2019 and 2020 so events during the pandemic year would be accurately compared to the same events/timing in previous year. All phases have the same number of days and number of weekdays. For the Population Decline period, the dates included the Friday before spring break through the last Sunday of spring break. Dates are from the Office of the Registrar at Penn State [2].

| **Year** | **Baseline** | **Population Decline** (Spring Break) | **Local Restrictions** | **Red** | **Yellow**  (corresponds to commencement) | **Green** |
| --- | --- | --- | --- | --- | --- | --- |
| 2019 | Feb 8 | March 1 | March 11 | March 22 | May 3 | May 24 |
| 2020 | Feb 14 | March 6 | March 18 | March 27 | May 8 | May 29 |

**Table S3. Summary of daily traffic data.** Estimates of daily means of vehicle counts using observed and predicted counts from best-fit GAM.

|  |  | **Mean daily traffic by Restriction Phase** | | |
| --- | --- | --- | --- | --- |
| **Weekday/ Weekend** | **Road type** | **County Red** | **County Yellow** | **County Green** |
| Weekday | Internal | 10772 | 13267 | 15309 |
| Weekday | Connector | 7397 | 9729 | 12425 |
| Weekend | Internal | 12678 | 10455 | 12083 |
| Weekend | Connector | 6367 | 6367 | 8113 |

**Supplementary Figures**

**Figure S1. Comparison of SafeGraph Points of Interests and Traffic Camera Locations.** Major roads are shown in light gray. We used a two-dimensional kernel density to estimate the POI density across Centre County (function *kde2d* in the *MASS* package [8]).

**
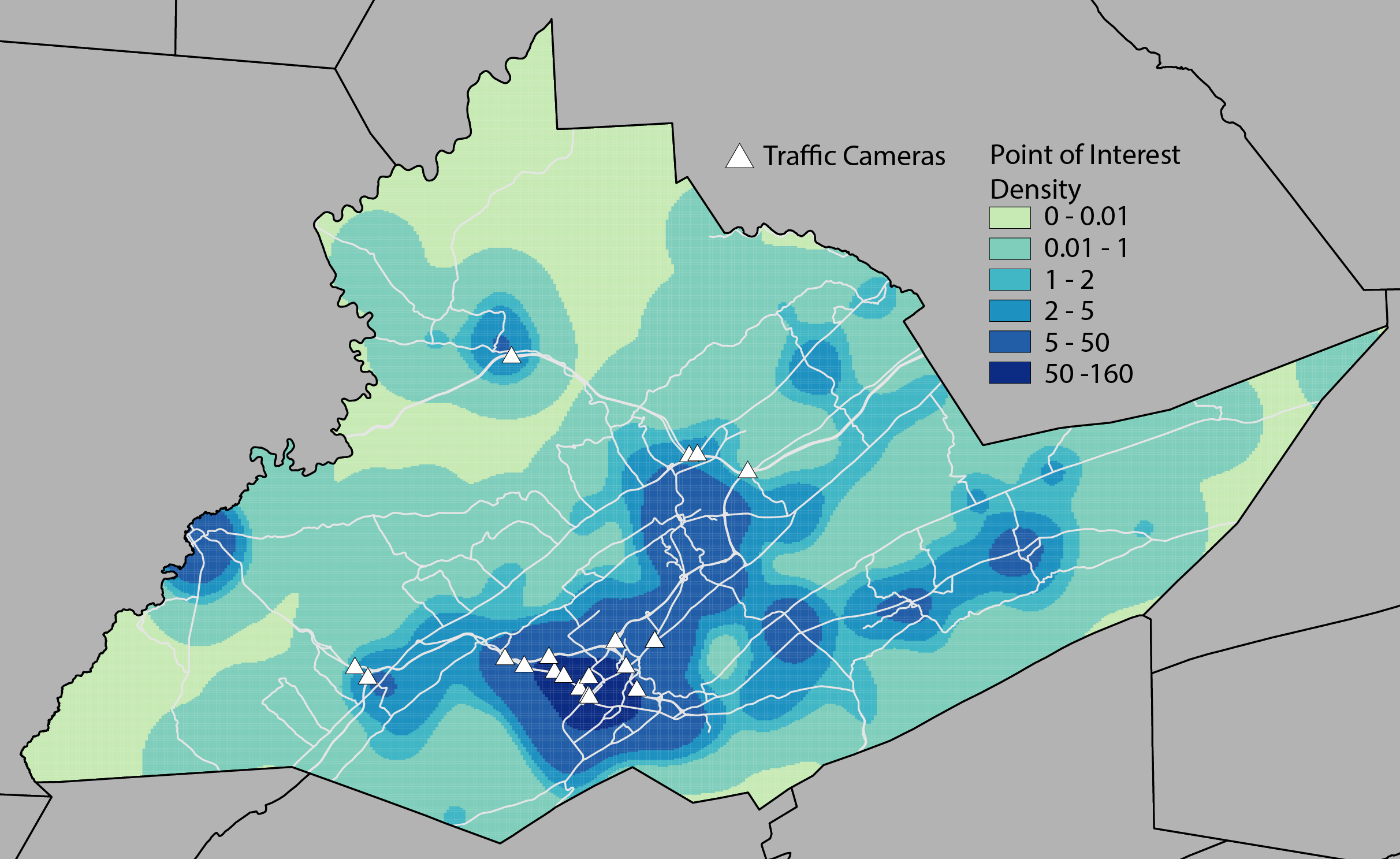
**

**Figure S2. Splines fit to hourly traffic volume by individual cameras.** The y- axes show the estimates of the best-fit GAM and x-axes show the hour of the day (range 0-23).

**Figure S3. Observed and predicted hourly count data per camera through time.** Among the 19 cameras and 108 days (49,248 camera-hours), only 5.96% of hours across all cameras were missing and had to be predicted. Predicted counts from the best-fit GAM were used to fill in gaps in observed data and are shown in open circles. Phases are indicated with vertical colored lines for Red, Yellow and Green Phases. Consistent with Fig 2 in the main text, shades of blue represent connector roads and orange shades represent internal roads. The range of the y-axis varies by camera.

**
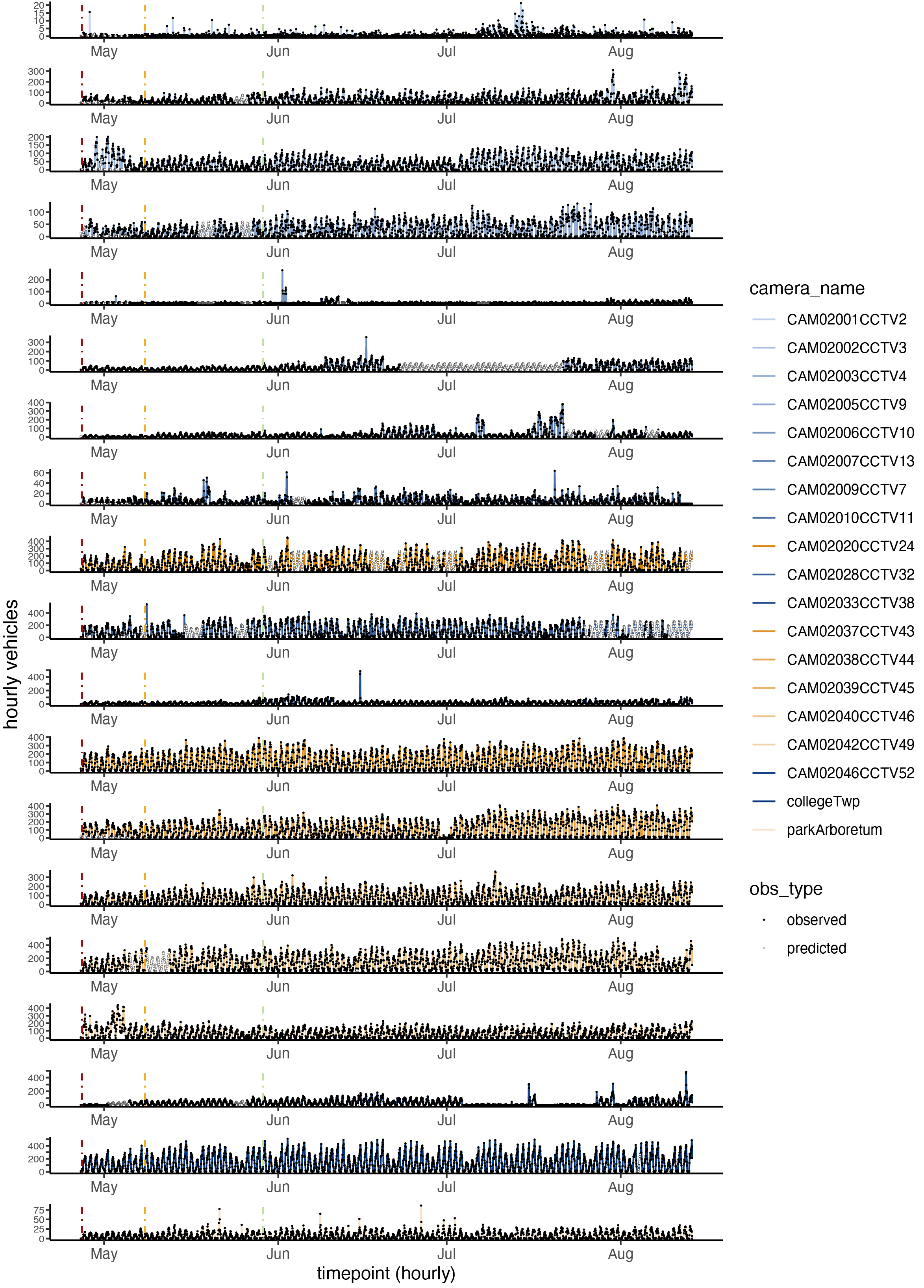
**

**Figure S4. Visit counts for Centre County, Spring and Summer 2019-2020**

Maximum mobile device visit counts from Centre County in 2019 reflected known academic calendar events and university or community events near or on PSU’s UP campus. This was not seen in 2020 when most events were cancelled and students were largely absent from the area.

**Supplementary References**

1. **Fuller T, Baker M**. *Coronavirus death in California came weeks before first known US death*. *NY Times*. 2020; Published online: 22 April 2020.

2. **PennState University Registrar**. *Academic Calendars*. 2020.

3. **World Health Organization,**. *WHO Director-General’s opening remarks at the media briefing on COVID-19*. 2020 Nov.

4. **Barron E**. *All Penn State classes to take place remotely beginning March 16*. *Penn State University*. 2020; Published online: 11 March 2020.

5. **Wolf T**. *Order of the Govenor of the Commonwealth of Pennsylvania regarding the closure of all businesses that are not life sustaining*. Commonwealth of Pennsylvania, 2020.

6. **Wolf T**. *Process to Reopen Pennsylvania*.

7. **Levine R**. *Order of the Secretary of the Pennsylvania Department of Health Requiring Universal Face Coverings*. 2020.

8. **Venables WN, Ripley BD**. *Modern applied statistics with S*. New York: Springer-Verlag, 2002.
